# NF-κB signaling in neoplastic transition from epithelial to mesenchymal phenotype

**DOI:** 10.1186/s12964-023-01207-z

**Published:** 2023-10-18

**Authors:** Amy Oh, Makayla Pardo, Anaelena Rodriguez, Connie Yu, Lisa Nguyen, Olin Liang, Anna Chorzalska, Patrycja M. Dubielecka

**Affiliations:** Division of Hematology/Oncology, Department of Medicine, Rhode Island Hospital, Warren Alpert Medical School of Brown University, One Hoppin St., Coro West, Suite 5.01, RI 02903 Providence, USA

**Keywords:** NF-κB signaling, Chronic inflammation, Cell–cell junctions, Cytoskeletal reorganization, Apical-basolateral polarity, Epithelial-to-mesenchymal transition, EMT, Metastasis

## Abstract

NF-κB transcription factors are critical regulators of innate and adaptive immunity and major mediators of inflammatory signaling. The NF-κB signaling is dysregulated in a significant number of cancers and drives malignant transformation through maintenance of constitutive pro-survival signaling and downregulation of apoptosis. Overactive NF-κB signaling results in overexpression of pro-inflammatory cytokines, chemokines and/or growth factors leading to accumulation of proliferative signals together with activation of innate and select adaptive immune cells. This state of chronic inflammation is now thought to be linked to induction of malignant transformation, angiogenesis, metastasis, subversion of adaptive immunity, and therapy resistance. Moreover, accumulating evidence indicates the involvement of NF-κB signaling in induction and maintenance of invasive phenotypes linked to epithelial to mesenchymal transition (EMT) and metastasis. In this review we summarize reported links of NF-κB signaling to sequential steps of transition from epithelial to mesenchymal phenotypes. Understanding the involvement of NF-κB in EMT regulation may contribute to formulating optimized therapeutic strategies in cancer.

Video Abstract

**Supplementary Information:**

The online version contains supplementary material available at 10.1186/s12964-023-01207-z.

## Introduction

Inflammation is now recognized as playing an important role in different stages of tumorigenesis, including initiation, promotion, malignant conversion, invasion, and metastasis, and NF-κB is one of the major factors linking inflammation and cancer [1]. Multiple observations have highlighted the aberrant or constitutive NF-κB activation in a number of human cancers, including lymphoma, liver, lung and breast cancers. Abnormal NF-κB activation is also driven by environmental stimuli commonly associated with carcinogenesis, such as tobacco and/or alcohol use, and irradiation. The major tumorigenic function of NF-κB has been linked to disturbed regulation of the transcription of targets associated with the cell cycle including cyclin D1/D2 and CDK 2/CDK6, and apoptosis, including cIAP1, XIAP and c-FLIP, resulting in abnormal cancer cell progression and the suppression of apoptosis respectively [2]. NF-κB activation was also reported to be involved in tumorigenic angiogenesis and tumor cell invasion [3]. Constitutively active NF-κB signaling results in secretion of major inflammatory cytokines or chemokines, including TNFα, IL-1 or IL-6, which, through a positive feedback loop, increase NF-κB activation, further contributing to uncontrolled growth and malignant transformation. Therefore, a better understanding of NF-κB and its association with tumor-promoting inflammation and anti-tumor immune suppression will likely facilitate the development and optimization of cancer prevention and treatment.

Epithelial to mesenchymal transition (EMT) is a process in which epithelial cells acquire mesenchymal phenotype and lose epithelial features. EMT involves a sequence of steps that include 1) loss of stable epithelial cell–cell junctions, 2) loss of apical–basal polarity and interactions with basement membrane, 3) cytoskeletal rearrangements leading to acquirement of fibroblast-like morphology and cytoarchitecture, 4) increased migratory capacity and 5) acquirement of invasive properties. EMT normally occurs during early embryonic development or during wound healing process in adults. EMT is also activated during carcinogenesis and is involved in cancerous expansion, metastasis, cancer recurrence and development of several types of fibrosis. It is important to emphasize that EMT is associated with phenotypic heterogeneity due to the often incomplete transition from epithelial to mesenchymal state, resulting in an array of intermediate states in which cells retain both epithelial and mesenchymal characteristics. These intermediate states are collectively named a state of epithelial-mesenchymal plasticity. The completion of EMT is typically accompanied by a switch in intermediate filament utilization from cytokeratins to vimentin. In the early 1990s, a number of transcription factors (TFs), including Slug, Snail, E47, Twist1, Zeb1 and Zeb2, were identified by means of their ability to induce EMT phenotypes and orchestrate the process. These EMT TFs control cell–cell adhesion, cell migration and degradation of the extracellular matrix. It also became apparent that activation and execution of EMT does not require permanent changes in DNA sequence and instead is fine-tuned by epigenetic regulators. Given the heterogeneity of EMT states and pleiotropy of observed intermediate phenotypes it has become clear that EMT state should be defined based on collective features including activity of core EMT TFs as well as morphological and cytological phenotypes [4]. In this review, in an effort to better understand how NF-κB-driven inflammation contributes to carcinogenesis, we attempt to comprehensively summarize the current knowledge of the involvement of the NF-κB signaling in the control of core EMT changes including cytoskeleton remodeling, loss of apical–basal cell polarity, cell–cell adhesion weakening and cell–matrix adhesion remodeling, acquisition of cell motility and basement membrane invasion. In this review, we approach the subject from cancer cell-centric view to highlight the role of NF-κB signaling in cancer cells undergoing transition. While NF-κB signaling plays an important role in modulating tumor microenvironment (reviewed in [5]), this is beyond the scope of this summary.

### NF-κB structure and pathway overview

#### The NF-κB family and structure

**Fig. 1:**
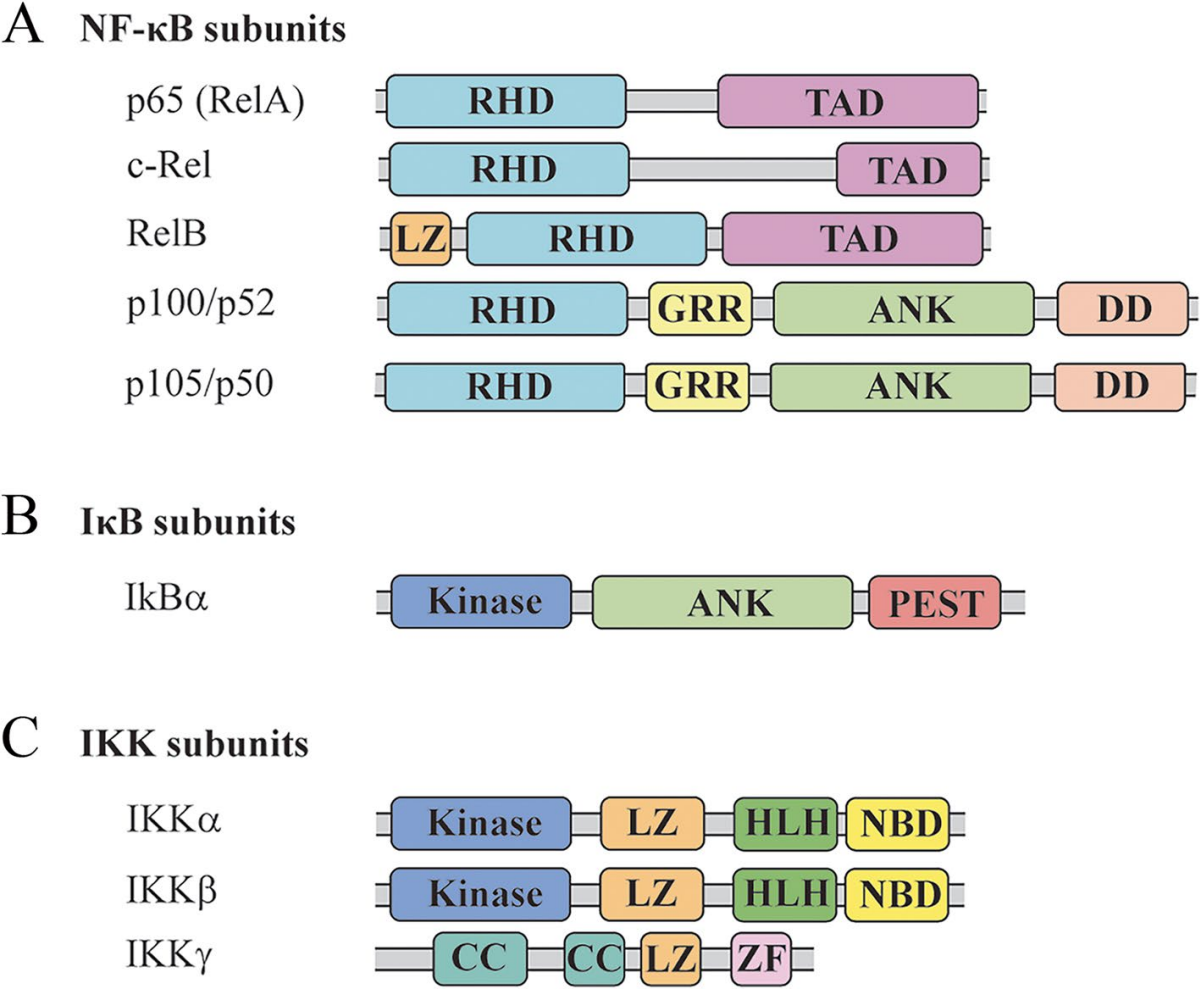
Schematic representation of NF-κB, Iκß, and IKK family members. (**A**) NF-κB family members share the RHD (Rel Homology Domain), important for DNA binding and dimerization. The functional domains of each subunit are indicated schematicaly: TAD = transcription activation domain; LZ = leucine zipper; GRR = glycine-rich domain; ANK = ankyrin repeats DD = death domain (**B**) Iκß family members share ANK domain that allows interaction with the RHD of NF-κB. Other indicated domains include PEST = proline/glutamic acid/serine/threonine-rich sequence. (**C**) The three IKK subunits are represented with domains that typify each protien: HLH = helix-loop-helix; NBD = NEMO-binding domian; CC = coiled-coil; ZF = zinc finger

The Nuclear Factor-κB (NF-κB) family of transcription factors regulates a large number of genes involved in a multitude of functions, including cell survival, proliferation, and immune responses. This family consists of five proteins—p65 (RelA), RelB, c-Rel, p50/p105 (NF-κB1), and p52/p100 (NF-κB2), all of which contain a highly conserved 300-residue long region, termed Rel Homology Domain (RHD) responsible for dimerization, DNA recognition, DNA binding and nuclear localization [6, 7] (Fig. 1A). The NF-κB family members can be further divided into two subgroups based on the sequences C-terminal to the RHD. One group, consisting of RelA, RelB, and c-Rel, contains a transactivating C-terminal region, while the other group, consisting of p50/p105 and p52/p100, on its C-terminus contains a structural motif, the death domain (DD). The members of each group can form inter- and intra-, homo- and hetero-dimers. Depending on the presence or absence of the transactivating domain, they function as either activators or repressors of transcription [8, 9]. The formation and stability of the NF-κB dimers is dependent on the sequence of amino acid residues in direct contact with each other, forming the interface, while amino acid residues outside of the interface modulate the local binding environment. The most abundant form in most cells, p50:p65(RelA) heterodimer, is one of the most stable dimers, whereas RelB homodimer does not exist in vivo due to the low stability of the RelB dimerization domain destabilized by non-interfacial amino acid residue interactions [10–12]. The first x-ray crystal structure of NF-κB p50 homodimers bound to DNA, resolved by Harrison and Sigler, showed that the RHD folds into two immunoglobulin-like domains [13, 14]. The N-terminus of one of the domains spans 160–200 amino acids and interacts with the major grove of DNA in base-specific manner, while the C-terminus of the other domain, being about one hundred amino acids in length, contributes to the hydrophobic residue-mediated dimerization, while interacting with DNA in nonspecific manner. Resolved NF-κB p50 homodimer-DNA complex provides evidence that the entire RHD scaffolding is required for the DNA recognition and interaction [6, 12, 15, 16]. The NF-κB dimers that translocate to the nucleus bind to decametric DNA sequence motifs containing the general consensus—GGGRNNYYCC (N denotes any nucleotide, R is for purine bases, and Y is for pyrimidine bases), known as κB sites. The p50 and p52 proteins prefer κB sites comprised of two GGGRN half-sites, separated by A/T base pair. The RelA, c-Rel and RelB proteins bind to κB sites containing two YYCC half-sites. The heterodimers (p50:RelA or p50/p52:RelB) show similar binding affinities to both types of κB sites. These mechanisms allow each hetero- or homodimer to mediate discrete cellular responses dependent on physiological contexts in response to numerous stimuli [10, 15, 17]. Importantly, however, NF-κBs are also able to bind to κB DNA sites with significant deviations from the consensus sequences. If the deviation occurs within the central region of the consensus sequences, the overall binding conformation remains the same, given the flexibility of the linker region, but the stability or the binding affinity, may change [12]. These unique features of NF-κBs structure and DNA binding ability allow NF-κB regulating the numerous genes and processes.

#### The IκB family and structure

In the absence of stimuli, NF-κB is normally sequestered in the cytosol through the interactions with the proteins of the IκB family. IκB proteins are a subfamily within the large Ankyrin Repeat Domain (ARD) containing superfamily and can be classified into three categories: classical IκB (IκBα, IκBβ, and IκBε), NF-κB precursors (NF-κB precursor p105 and p100), and nuclear IκB (IκBζ, Bcl-3 and IκBNS). Classical IκBs contain phosphorylation and poly-ubiquitination sites at the N-terminus and the NF-κB:DNA complex disrupting PEST region, composed of proline, glutamic acid, serine, and threonine, at the C-terminus (Fig. 1B). IκBα contains a nuclear export signal that mediates the localization of the NF-κB:IκBα complex to the cytoplasm [12]. The X-ray crystal structure of IκBα:NF-κB p50/65 heterodimer complex shows the conserved mode of IκB binding: the ankyrin repeat of the IκBα runs in the antiparallel direction, curves towards, and binds to the NF-κB heterodimer in a “cupped hand” manner, inhibiting its binding to DNA. The ankyrin repeats 1 and 2 form hydrophobic contact with the nuclear localization signal located at the dimer’s C-terminal domain, while ankyrin repeats interact with the RHD at the same terminus. The acidic property of the ankyrin repeats and PEST region repels the positively charged N-terminal domain of the p65 subunit, which undergoes significant conformation change into a “locked” form that completely masks the nuclear localization signal. A similar binding pattern is observed in IκBβ:NF-κB p50/65 heterodimer, but with less dependence on the interaction between the N-terminal domain of the NF-κB dimer. IκBβ does not directly bind to the p65 subunit N-terminal domain, leaving the nuclear localization signal and the DNA-binding domain free to bind to DNA. Consequently, studies have shown that the IκBβ:NF-κB p50/65 complex is found in both cytoplasm and nucleus, while the IκBα:NF-κB p50/65 complex is exclusively located in the cytoplasm [8, 16, 18]. A series of biophysical experiments, including single-molecule fluorescence resonance energy transfer (FRET), have shown that classical IκBs are inherently unstable and remain incompletely folded in their free states, subjected to steady-state, signal-independent degradation by 20 s proteasome and the C-terminal PEST of IκBs. Upon binding to NF-κB, IκBs switch from the extended to compact form and become stable until the PEST region is degraded in signal-dependent manner through phosphorylation of the N-terminal response domain [10, 19]. The nonclassical IκB proteins, NF-κB precursors, are similar to the classical IκB proteins in two major aspects: they contain ankyrin repeats and mediate the gene expression level through the interaction with NF-κB dimers. Unlike the classical IκB proteins that form 1:1 complex with the NF-κBs, the nonclassical IκB proteins are capable of binding to more than one NF-κB dimers through their oligomerization domain, forming a multimeric complex. They also show different binding affinities for the NF-κB members: classical IκBs bind to NF-κB dimers that contain at least one p65 or c-Rel subunits, while nonclassical IκBs are limited to binding p50 or 52 homodimers. Given the variation in the binding affinities, IκB proteins can function as modulators of NF-κB dimerization, determining the prevalence of the NF-κB dimers, which may play an important role in the NF-κB transcriptional specificity [12, 16]. Finally, nuclear IκB proteins do not contain the N-terminal signal-dependent phosphorylation sites, or the C-terminal PEST region, but they are still classified as IκB family, considering that they have ankyrin repeats and are capable of binding to NF-κB subunits, namely p50 homodimers only. They are known to play an important role in controlling gene expression and immune homeostasis, as for example some experiments have demonstrated that mice were not capable of producing IL-6 response to the LPS treatment in the absence of nuclear IκB proteins [12]. In sum, all three classes of IκBs add to the complexity of NF-κB transcriptional specificity.

#### The IKK complex

IKK family, comprised of IKKα (IKK1), IKKβ (IKK2), and IKKγ (also known as NEMO), functions as a converging point for the majority of the NF-κB activating signaling pathways. NEMO, the key regulatory non-enzymatic scaffold protein, is required for the catalytical subunits to fully gain the inducible kinase activity, although the exact oligomerization state of the IKK complex is poorly understood. The IKK catalytic subunits are organized into five distinct parts: the N-terminal kinase domain, followed by the ubiquitin-homology region, leucine zipper and helix-to-helix motifs in the center responsible for dimerization, and finally the serine-rich region and NEMO-binding site at the C-terminus (Fig. 1C). The regions outside of the kinase domain mediate recognition and exact positioning of the IκB substrates, as studies have shown that IKK complex loses its binding specificity in the absence of leucine zipper and helix-to-helix motif regions [12, 16, 20]. The catalytic subunits exist in dimeric structure, forming homo- or hetero-dimers that resemble a pair of scissors. The kinase domain of the two monomers is located far from each other, incapable of stimulating intradimer trans-autophosphorylation, while they interact and activate the kinase domain of the neighboring dimers through NEMO-mediated ubiquitin chain network. The mutagenesis studies of IKKβ suggest that the dimerization of the kinase domain is necessary for NEMO binding and recruitment of the IκBα substrate [18, 20, 21]. While multiple X-ray structures of the fragments of NEMO have been reported, the full-length protein structure of NEMO subunit remains unknown [18, 20]. Assembling the structures of the isolated domains shows that NEMO is composed of the symmetrical helical-shaped (except the zinc-finger region) dimers, containing two helices (HLX1 and HLX2), two coiled-coil domains (CC1 and CC2) in configuration HLX1, CC1, HLX2, CC2, followed by leucine-zipper domain and C-terminal zinc-finger (ZF) region (Fig. 1C). The first two regions, HLX1 and CC1, form the NEMO binding site for IKKα/β, and the ubiquitin-binding motif is located on the C-terminus and encompasses leucine-zipper and ZF regions. Chemical cross-linking and equilibrium sedimentation analyses suggest that NEMO dimers can interact with IKKβ homodimers, forming helix-shaped hetero-tetramer. The two NEMO molecules interact with each other at the N- and C-terminus, while the two IKKβ molecules interact with each NEMO molecule individually, without interacting with each other. Stoichiometrically, this hetero tetramer can bind to two IKKα and IKKβ molecules, contributing to the IKK trans-autophosphorylation [18, 20]. The NEMO plays a crucial role in the NF-κB cascade for its ability to recognize and bind to the poly-ubiquitinated sites (both N-terminal methionine-linked di-ubiquitin and lysine 63-linked polyubiquitin) on the proteins involved in the NF-κB activation, functioning as an adaptor linking the catalytic subunits and other receptor signaling molecules [18, 22]*.*

#### The NF-κB activation cascade

**Fig. 2:**
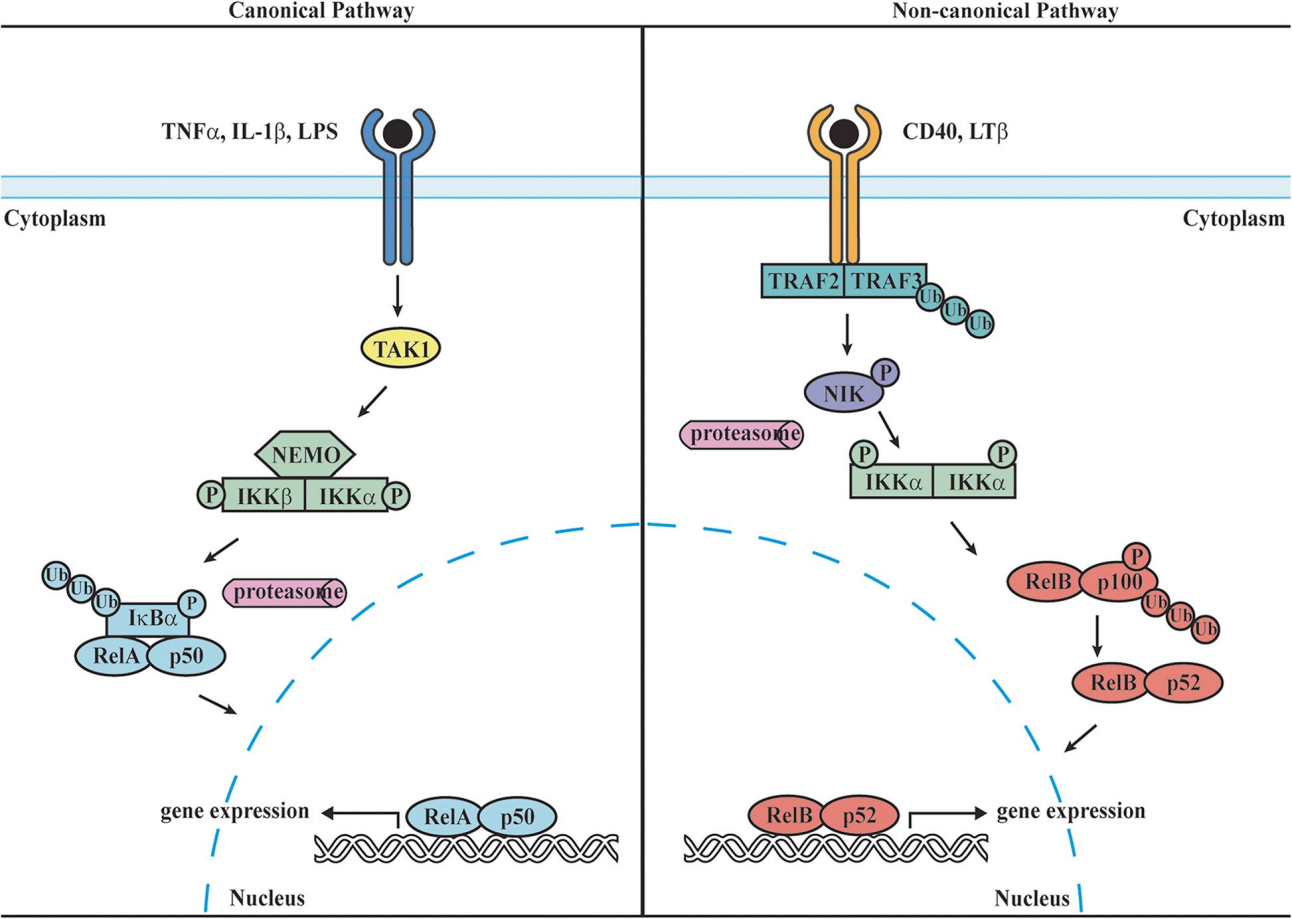
Activation of canonical and non-canonical NF-κB signaling. The activation of the canonical pathway is induced by proinflammatory cytokines (e.g., IL-1ß, TNFα) binding to their respective receptors on the cell surface. This triggers the activation of TAK1 (transforming growth factor ß-activated kinase 1), which in turn activates the IKK complex, consisting of the regulatory NEMO and the catalytic subunits IKKα and IKKß. The activated IKK complex then phosphorylates the IκB protein, triggering Iκß ubiquitination and proteasomal degradation. The classical NF-κB dimers are released and translocate to the nucleus to regulate gene expression. Unlike the canonical pathway, the non-canonical pathway is activated by a distinct set of stimuli, activating the TNFR superfamily, which results in the stabilization and accumulation of NIK kinase. Increased NIK protein level phosphorylates IKKα, which in turn phosphorylates p100 protein, leading to partial degradation and conversion into the active p52 subunit. The p52 subunit forms a heterodimer with RelB, which translocate to the nucleus and activates the transcription of target genes

In response to immune and stress stimuli, NF-κB becomes activated via two major pathways, canonical and noncanonical (Fig. 2). The canonical activation pathway involves signal-induced proteolysis of the IκBs, particularly IκBα, regulated by IκB kinases (IKKs). Upon activation by pro-inflammatory signals such as cytokines or pathogen-associated molecular patterns (PAMPs), IKKs phosphorylate the two serine residues in the N-terminal signal receiving domain of IκBs, leading to the poly-ubiquitination of the adjacent lysine residues. This results in degradation of IκBs in the proteasome and freeing of NF-κBs. The freed p50-containing NF-κB dimers, the most common form being p50:RelA and p50:c-Rel heterodimers, translocate to the nucleus and bind to their target promoter sites. The canonical pathway is known to play a critical role in regulating immune responses, including lymphocyte activation and differentiation, innate immunity, and inflammation [18, 23]. A selective set of differentiating and developmental stimuli, largely belonging to the tumor necrosis factor receptor (TNFR) superfamily, are known to activate the non-canonical pathway. It is characterized by the processing of the NF-κB precursor protein p100 through the phosphorylation of its C-terminal serine residues by NF-κB inducing kinase (NIK) and/or IKKα. Increasing evidence suggests the involvement of the DD of p100 in the processing, as its removal leads to constitutive processing. The processed p52 subunit then dimerizes with RelB to enter the nucleus to regulate transcription of genes involved in lymphoid organ development, B cell maturation, osteoclast differentiation and broadly autoimmune and inflammatory responses [1, 23, 24]. The activation of NF-κB through the canonical pathway is rapid but transient and is terminated by the NF-κB-mediated re-synthesis of IκB proteins, which disrupts the NF-κB:DNA binding and results in export of the transcription factors back to the cytosol. In contrast, non-canonical activation is slow due to its dependence on the ubiquitination-regulated stabilization of NIK [17, 20].

#### The receptor-induced signaling cascade

Several receptor-induced IKK activation cascades have been identified, in which TNFR-and toll-like receptor/interleukin-1 receptor (TLR/IL-1R) superfamily-induced activations have been extensively studied. The TNFR-induced IKK activation pathway begins with extracellular ligands binding to TNFR, which recruits TNFR-associated factors (TRAFs) directly or through adaptor proteins. TRAFs contain N-terminal RING finger domain, followed by ZF, and C-terminal CC and TRAF-C region. Typically, the N-terminal region is responsible for dimerization and mediates lysine 63-linked polyubiquitination, while C-terminal is involved in trimerization and interactions with the receptor and adaptor proteins. Each termini provides a scaffold for TRAF aggregation and higher-order oligomerization and locally concentrates all of the associated signaling proteins, which facilitate the autoubiquitination, polyubiquitination, and downstream signaling. cIAP1/2, recruited by the CC region of TRAFs, drives the polyubiquitination of multiple proteins, such as receptor-interacting serine/threonine-protein kinase 1 (RIPK1), NIK, TRAF2, which leads to recruitment of downstream proteins, including NEMO and ubiquitin ligase. The binding of NEMO to the ubiquitin chain complex initiates the IKK activation, either through inducing the conformational changes or positioning IKK to have it exposed to phosphorylation by upstream kinases in the complex [2, 18, 20, 21]. The TLR and IL-1 superfamily shares a common Toll/1L-1R (TIR) intracellular domain, activating overlapping downstream cellular signals. The primary adaptor protein recruited by the TIR domain is MyD88, a member of the DD superfamily. The death domain of MyD88 oligomerizes with the IL-1R-associated kinase (IRAK) family members, IRAK4, IRAK1 and IRAK2, to form a complex termed *myddosom*e. The IRAK4 initiates the auto-phosphorylation of itself and facilitates the phosphorylation of the other IRAK members in the complex. Next, the phosphorylated IRAK1 and IRAK2 recruit TRAF6, a ubiquitin E3 ligase that catalyzes lysine 63-mediated autoubiquitination and polyubiquitination in the signaling pathway, inducing the IKK activation followed by phosphorylation and ubiquitination of IκBs resulting in activation of NF-κB [16, 18, 23].

### The NF-κB role in EMT

**Fig. 3:**
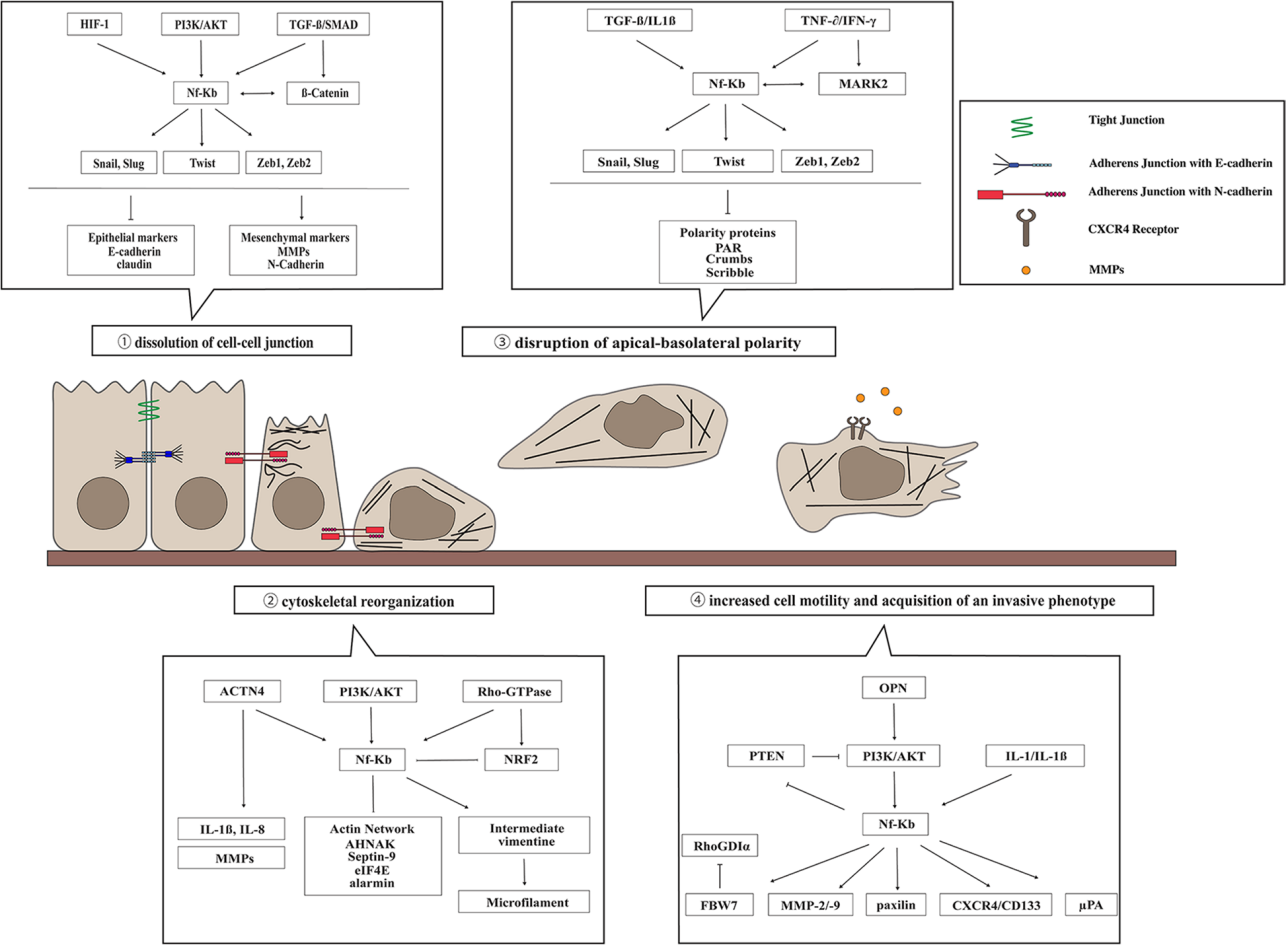
Schematic diagram of the EMT transition stages. Epithelial-mesenchymal transition (EMT) is a dynamic process in which epithelial cells undergo a transition into a mesenchymal state, leading to changes in their morphology, function, and behavior. The early-stage cells display epithelial features: apical-basal polarity is present, epithelial-associated proteins are expressed, and tight and adherens junctions hold the cells together. EMT involves a sequence of steps that starts with the loss of stable epithelial cell–cell junction, leading to loss of cell polarity and adhesion. The following remodeling of the cytoskeleton results in extensive rearrangement of actin filaments and microtubules, with cells gaining mesenchymal-like morphology and cytoarchitecture. The overexpression of regulators of EMT, such as transcription factors Snail, Slug, Twist, and Zeb1/2, leads to changes in gene expression, activating those associated with mesenchymal cell characteristics, including N-cadherin, MMPs, and vimentin. The mesenchymal cells exhibit increased migratory capacity and acquire invasive behavior, allowing them to disseminate into surrounding tissues. The major steps of EMT are highlighted with specific link to NF-κB signaling outlined

The NF-κB signaling has been implicated in multiple aspects of oncogenesis, including pro-inflammatory signaling, cell differentiation, migration, and tissue remodeling. Previous research has demonstrated constitutive activity of NF-κB, or mutations in genes encoding upstream regulators of NF-κB, in a significant number of human cancers, especially those of immune cell origin, such as leukemias and lymphomas. Recently, studies further suggested that NF-κB plays an essential role in induction and maintenance of invasive phenotypes in cancer, including EMT and metastasis, however the detailed mechanisms underlying NF-κB links to EMT remain unclear. Therefore, herein we summarize the current understanding of the involvement of NF-κB in EMT (Fig. 3), as delineating this relationship has a potential to facilitate the development and optimization of therapeutic strategies in cancer.

#### NF-κB and dissolution of cell–cell junctions

Research investigating the relationship between NF-κB and EMT-associated dissolution of intercellular junctions focuses mainly on epithelial cadherin (E-cadherin), not only because it is a major epithelial marker and its decreased expression is considered a major hallmark of EMT, but also due to its function as a transmembrane protein and a major epithelial calcium-dependent cell adhesion molecule. Homophilic binding between E-cadherins of adjacent cells forms the basis of the epithelial cell–cell contacts—adherens junctions (AJs), which, together with other molecules, form an adhesion junctional complex. There are several reports highlighting the link between NF-κB and E-cadherin in AJs. Tripathi et al. [25] demonstrated that Rho GTPase-activating protein (RhoGAP) - Deleted in Liver Cancer 1 (DLC1), the downregulation of which is associated with prostate carcinoma (PCA), stabilizes AJs in PCA cell lines through binding to E-cadherin and as such has an inhibitory effect on NF-κB activation. Solanas et al. [26] also found that NF-κB as well as transcriptional activator β-catenin, both associate with E-cadherin at AJs. This interaction stabilizes AJs and has an inhibitory effect on the transcriptional activity of NF-κB and β-catenin, suppressing the expression of various mesenchymal markers central to EMT. Kuphal et al. [27] found that the constitutive activation of NF-κB led to decreased expression of E-cadherin within malignant melanoma cells, leading to the concomitant increase in free cytoplasmic β-catenin further leading to the p38 MAPK-mediated activation of NF-κB. Zipper-interacting protein kinase (ZIPK) or Death-Associated Protein Kinase 3 (DAPK3), is a part of the death-associated protein kinase family regulating apoptosis. Li et al. [28] found that the elevated levels of ZIPK in gastric carcinoma (GC) cells are linked to increased expression of Snail and Slug, decreased expression of E-cadherin and overexpression of mesenchymal markers and dissolution of intercellular junctions. Furthermore, it was demonstrated that the increased activation of Akt mediated by ZIPK does not lead to increased activation of PI3K/Akt/GSK3β but rather the PI3K/Akt/ΙΚΚ/IκBα/NF-κB signaling axis, presumably leading to the significantly increased expression of Snail and Slug and induction of the downstream EMT phenotype. In another study, Gao et al. [29] investigated the role of insulin-like growth factor-binding protein 2 (IGFBP2) in pancreatic ductal adenocarcinoma (PDAC). They found that IGFBP2 overexpression resulted in significantly increased expression of Snail, decreased expression of E-cadherin at intercellular junctions, increased expression of mesenchymal markers, nuclear translocation and overactivation of NF-κB and dissolution of intercellular junctions. Furthermore, Gao et al. demonstrated that IGFBP2-induced EMT is dependent on the increased activation of NF-κB, which they found to be linked to increased activation of the PI3K/Akt/ΙΚΚ/IκBα/NF-κB signaling axis. Cichon and Radisky [30] looked closer at NF-κB signaling to elucidate the molecular mechanism underlying matrix metalloproteinase 3 (MMP3)-induced EMT. MMP3 was shown to induce EMT in mammary epithelial cells via increased expression of Rac1b, an activated splice variant of Rac1 Rho GTPase, and subsequent stimulation of ROS production. Cichon and Radisky verified that MMP-3/Rac1b/ROS induces EMT in mammary epithelial cells. Presence of MMP3 resulted in significantly increased expression of Snail, significant activation of a tumorigenic transcriptional profile, including alterations of transcripts related to intercellular adhesion, mesenchymal morphology, and dissolution of intercellular junctions. They determined that MMP3/Rac1b/ROS-induced EMT requires ROS-dependent activation of NF-κB and that the activation of NF-κB results in upregulation of Snail via direct binding of NF-κB to its promoter. Cheng et al. [31] determined the status of EMT transcription factors Twist, Zeb1, and Zeb2 alongside Snail as they investigated the previously established association of tumor hypoxia, the expression of hypoxia-inducible factor-1 (HIF-1) and the constitutive activation of NF-κB with the development of pancreatic cancer (PC). They found that hypoxic conditions or overexpression of HIF-1α led to increased NF-κB activity, resulting in upregulation of Twist but not Snail, Zeb1, or Zeb2. Although the findings of Cheng et al. continue to corroborate the general relation demonstrated by the findings of Li et al., Gao et al., and Cichon and Radisky, in which increased activation of NF-κB leads to an EMT phenotype including the dissolution of intercellular junctions, the findings of Cheng et al. provide nuance to the specific EMT transcription factor regulation by NF-κB, which can be cell type dependent. Indeed, Chua et al. [32] found that mammary epithelial cells treated with TNFα or transduced to overexpress a constitutively active form of the p65 subunit of NF-κB, undergo EMT driven by an increased expression of Zeb1 and Zeb2 but not Snail or Slug, further highlighting potential cell type specific context of the effect of NF-κB on EMT TFs. Besides the PI3K/Akt, the TGF-β1/Smad signaling pathway has also been implicated in the EMT-associated increased activation of NF-κB via the canonical ΙΚΚ/IκBα/NF-κB axis. Transforming growth factor-β1 (TGF-β1) is a key mediator of EMT that has been shown to induce decreased expression of E-cadherin, increased expression of mesenchymal markers, and gain of mesenchymal morphology highlighted by dissolution of intercellular junctions. Lee et al. [33] found that TGF-β1 treatment of breast cancer (BC) cells results in significant activation of IκBα and NF-κB, significant increases in Snail and Slug expression and significant decreases in levels of E-cadherin and development of EMT phenotype. In summary, these findings indicate that the dissolution of intercellular junctions, mainly through downregulation of E-cadherin, is mechanistically linked to increased NF-κB activity during EMT, both through increasing the translocation of NF-κB to the nucleus and/or by increasing the overall expression and/or activity of NF-κB (Fig. 3). Further research is required to detail the mechanistic nuances of the effect of NF-κB signaling on the stability of AJ in the cell type specific manner.

#### NF-κB and cytoskeletal reorganization

**Fig. 4:**
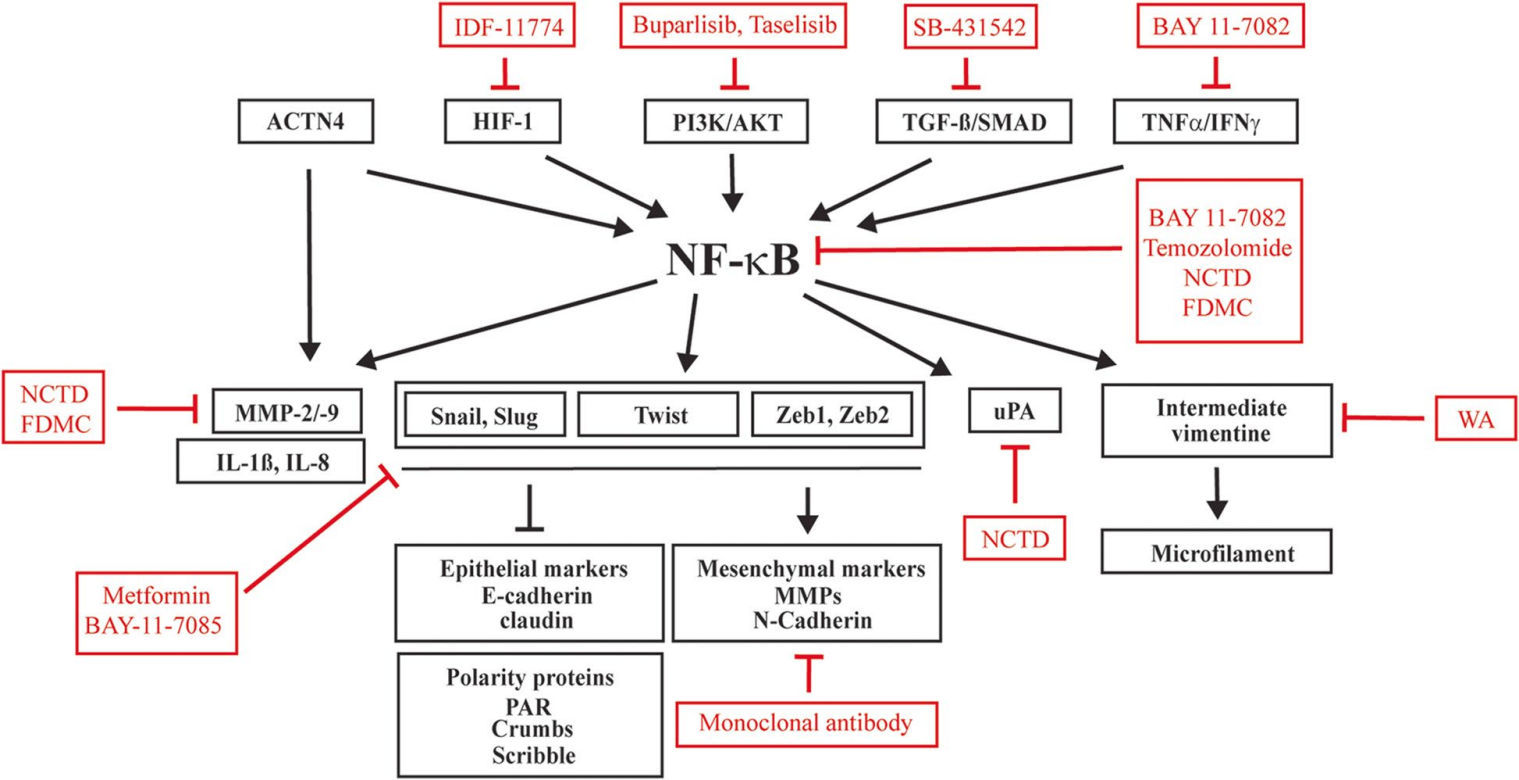
Potential therapeutic strategies for targeting EMT. Several strategies exist for targeted EMT treatment: inhibition of EMT induction by blocking upstream signaling pathways, suppression of EMT-associated transcription factors or blocking the colonization of mesenchymal cells. Here we highlight some of the dugs that target specific signaling intersections between NF-κB and EMT. Norcantharidin (NCTD), Farnesyl dimethyl chromanol (FDMC), Withaferin A (WA)

The acquisition of mesenchymal-like phenotype during EMT leads to enhanced migratory and invasive abilities of cancer cells, which are mediated by cytoskeletal reorganization (Fig. 3). The crucial role of the cytoskeleton in the EMT was first proposed by Shankar et al. [34], who demonstrated that the inhibition of cancer-associated proteins resulted in the reduction of actin dynamics. Further research extensively examined dynamic reorganizations of the cytoskeleton required for EMT. Loss or inhibition of components of the actin network, specifically AHNAK (desmoyokin), septin-9, Eukaryotic Translation Initiation Factor 4E (eIF4E), or alarmin S100A11 led to reduction of formation of podosomes, invadopodia, filopodia and lamellipodia, resulting in reduced migration and invasion, and a reversal of EMT. Dinicola et al. [35] showed that treatment with inositol led to inhibition of PI3K and phosphorylation of Akt, which negatively impacted NF-κB and Snail leading to increased levels of E-cadherin, redistribution of β-catenin and reduction of membrane protrusions and cell motility. Avci et al. [36] found that co-treatment of glioblastoma cells with an NF-κB inhibitor that inhibits TNFα-induced IκBα phosphorylation—BAY 11–7082 and alkylating agent Temozolomide resulted in significant reduction in cell viability, suppressed NF-κB signaling, and enhanced apoptosis via actin skeleton modulation (Fig. 4). Aksenova et al. [37] investigated the transcriptional effect of actin-binding protein alpha-actinin 4 (ACTN4) on the RelA subunit of NF-κB. It was found that ACTN4 overexpression leads to co-activation of RelA, upregulation of matrix metalloproteinases MMP3 and MMP1, and enhancement of cellular motility. Zhao et al. [38] found that ACTN4 promotes expression of NF-κB target genes such as IL-1β and IL-8. In sum, NF-κB activity was shown to be central for cellular motility and invasiveness. Additionally, three major Rho GTPases – Rac1, RhoA and Cdc42, central for regulation of actin polymerization in cells, were found to be required for NF-κB transcriptional activity and pathway activation [39]. Cuadrado et al. [40] reported that Rac1 activates both the nuclear factor-like 2 (NRF2) pathway and NF-κB activity, indicating that Rac1 may also influence inflammation by coordinating activity of NF-κB and NRF2 transcription factors. The RhoA–NF-κB interaction has been shown to be important in cytokine-activated NF-κB processes, such as those induced by tumor necrosis factor α (TNFα), whereas Rac1 is important for activating the NF-κB response downstream of integrins. Detailed involvement of Rho-GTPases in NF-κB signaling is reviewed in Tong et al. [41]. Homeostasis of the cytoskeleton depends on balanced interactions between its filamentous components—actin filaments, intermediate filaments, and microtubules. Intermediate filaments support the plasma membrane and help maintain cell shape. During EMT, intermediate filaments become vimentin enriched [42]. Shaedel et al. [43] shown that vimentin-enriched intermediate filaments stabilize microtubules against depolymerization contributing to enhanced migration, contractility, and resistance to mechanical stress in EMT. In the orthotopic model of pancreatic cancer Nomura et al. [44] showed that the cells pretreated with NF-κB inhibitor—BAY 11–7085 together with MYC-inhibitor—minnelide, showed decreased expression of tumor EMT-associated genes, such as Snail1/2, Zeb1, vimentin, MMP9 or N-cadherin. Treatment with these inhibitors subsequently led to decreased tumor volume and restoration of cell-to-cell junctions (Fig. 4). In conclusion, due to its intrinsic association with EMT phenotype switch, the cytoskeleton emerges as a particularly attractive therapeutic target. To date, however, no studies have systematically examined the synergistic cross-communication between the cytoskeleton components and NF-κB signaling and their effect on EMT. Therefore, a better understanding of this crosstalk and its potential pharmacological validity is required.

#### NF-κB and disruption of apical-basolateral polarity

Fundamental for homeostasis of epithelial cells—cell polarity—refers to the intrinsic asymmetric distribution of molecular components and general cellular structure and is a fundamental for homeostasis of epithelial cells [45, 46]. Cell polarity plays an essential role in maintaining tissue homeostasis and is linked to tumor suppression [47, 48]. Cell polarity spatially governs the signaling pathways within the cells, which helps cells process and integrate changes in the microenvironment to control morphology, differentiation, and motility, while regulating and maintaining the apical junctional complex (AJC), including tight junctions (TJs) and adherens junctions (AJs). Given its ability to mediate many functions in cells and tissues, the apical-basolateral polarity is considered a gatekeeper for tumor development and metastasis. Consequently, the loss of apical-basolateral polarity, which disrupts cell–cell communication and the ability to organize into tissue structure contributes to the EMT (Fig. 3). The cell polarity along the apical-basolateral axis is regulated by three evolutionary conserved protein complexes: PAR (PAR3, PAR6, and atypical protein kinase Cs (aPKCs)), Crumbs (Crumbs, PATJ, Stardust) and Scribble (Scribble, discs large (DLG), and lethal giant larvae (Lgl)). The PAR and Crumbs complexes together define the apical compartment, whereas the Scribble complex establishes the basolateral identity of the membrane [49]. The functions of these polarity regulating complexes are guided by phosphorylation and key protein–protein interactions between the components at the apical-lateral border [48]. Many studies have shed light on the mechanism underlying the EMT-mediated loss of cell polarity, which links EMT transcriptional regulators to the repression of cell polarity determinants. EMT TFs can directly target components of cell polarity regulatory complexes and can antagonize the expression of the polarity proteins by directly binding to the components of the complexes. For example, gene profiling screening and promoter analysis of Zeb1 showed that Crumbs3, Lgl2, and Pals1-associated TJ protein are all repressed by Zeb1 in breast cancer cell lines or colorectal carcinoma cells [50–52]. EMT-linked repressors can alternatively control the functions of the polarity proteins via non- or post-transcriptional mechanisms. The reduced expression of the Par polarity complex was observed after TGF-β treatment, where TGF-β interacts directly with and phosphorylates Par6, which leads to destabilization of the protein [46, 53]. Taken together, these observations highlight the existence of complex regulatory networks, in which different EMT-associated repressors act cooperatively to disrupt regulators of apical-basolateral polarity and TJs, that converge to the loss of cell polarity necessary for cells to undergo EMT. Through microarray analysis performed on breast cancerous and non-cancerous cells, forming less and more organized spheres in 3D culture respectively, Becker-Weimann and colleagues showed that NF-κB regulates the switch in polarity [54]. Sets of genes involved in the positive regulation of IKK and NF-κB cascade, including NF-κB itself, were upregulated in the disorganized spheres. The polarity was restored upon IKK inhibition. These results pointed to NF-κB as an important regulator of the induction of a disorganized phenotype in malignant breast cancer cells. Patients who receive peritoneal dialysis for renal disease often experience inflammation and injury to the peritoneum. In these patients, the mesothelial cells (MCs) of the peritoneum undergo changes that are similar to the epithelial-mesenchymal transition. This EMT-like phenotype, including the loss of cell–cell junctions, cell scattering and a spindled fibroblastic phenotype, a decrease in E-cadherin expression as well as an upregulation of the mesenchymal markers, fibronectin and N-cadherin, can be stimulated by both the peritonitis seen in peritoneal dialysis patients or with TGF-β and IL-1β treatment. The treatment also induced the nuclear translocation of NF-κB, and the observed phenotype in MC was reverted by the treatment with the IκBα super repressor, thereby establishing a link between NF-κB and the resulting EMT phenotypes [55]. EMT observed in head and neck squamous cell carcinoma (HNSCC) was shown to be induced by an inflammatory microenvironment, specifically the secretion of TNFα which activates the NF-κB pathway. When HNSCC cells were primed with TNFα, EMT phenotypes—including increase in cell area, a decrease in the number of neighboring cells, decrease in the intercellular contact and overexpression of markers characteristic of EMT—N-cadherin, vimentin and Snail/Slug, were noted [56]. These phenotypes were inhibited by siRNA knockdown of RelA, further confirming involvement of TNFα/NF-κB signaling axis in EMT phenotypes. Breast cancer stem cells (BCSCs) display resistance to chemotherapy and increased metastasis [57]. These cells displayed upregulation of AXL, a member of the TAM (Tyro3, Axl, and Mer) receptor tyrosine kinases, decrease in E-cadherin expression and an increase in N-cadherin, Snail and Slug as well as increase in NF-κB activity. In *C. elegans,* Microtubule Affinity Regulating Kinase 2—MARK2 helps to generate apico-basal polarity. The polarity complex including atypical PKC (aPKC) phosphorylates MARK2 at Thr595 to inhibit its activity so it can be released from the membrane. MARK2 then phosphorylates Par3, releasing it from the polarity complex, which results in changes in Par3 cytoplasmic distribution and changes in cellular polarity. It was shown that stimulation of NF-κB/IFN by TNFα and IFNγ leads to the downregulation of aPKC in intestinal epithelial cells resulting in an increase in NF-κB activity and chronic inflammation linked to elevated expression of IL-8 and CXCL1 [58]. All these observations suggest that NF-κB is linked to EMT-induced downregulation of polarity genes, driving the loss of apical-basolateral polarity. Further detailing the mechanistic link between the pathways regulating cell polarity and NF-κB signaling has the potential to inform new approaches to targeting overactive NF-κB and loss of cell polarity.

#### NF-κB and increased cell motility and acquisition of an invasive phenotype

EMT induction in cells results in an increase in cell motility and acquisition of an invasive phenotype facilitating metastatic progression and cancer recurrences. As discussed above, NF-κB has been recognized as being involved in the EMT process and playing a significant role in modulating the characteristic properties of EMT by regulating the expression of EMT-associated genes transcriptionally and post-transcriptionally. Dysregulation of the PTEN/PI3K/AKT pathway and CXCL12/CXCR4 axis in addition to activation of the metalloproteinase family are often cited as major contributors to the process of acquiring an invasive phenotype (Fig. 3), and number of studies have reported NF-κB contribution to these alterations.

#### PTEN/PI3K/AKT and NF-κB

Hyperactivation of the PI3K pathway has a well-established role in cancer progression and metastasis [59, 60], however little is known about the interplay between the PI3K/AKT and NF-κB pathways and their combined effects on cell motility and migration. Several recent reports have provided evidence of the emerging role of phosphatase and tensin homolog (PTEN) and its interaction with the NF-κB pathway in mediating the cancer cell invasion. It has been established that PTEN regulates cell apoptosis and survival signaling via PI3K/Akt pathway by dephosphorylating phosphatidylinositol, lipid second messenger produced by PI3K that acts downstream to activate Akt [61, 62]. The repression of the PI3K/Akt pathway by PTEN, in turn, inhibits its downstream molecules, including NF-κB. Interestingly however, recent studies reported that PTEN expression can be suppressed by the activation of RelA that is in turn suppressing PTEN promoter activity. CBP and p300, among others, are transcriptional co-activators of PTEN promoter. NF-κB was shown to competitively bind and sequester CBP/p300 [63]. This antagonistic coupling of NF-κB and PTEN controls the expression of many downstream effectors that drive cancer cell invasion and metastasis including F-Box and WD40 Domain Protein 7 (FBW7), RhoGDIα and paxillin. RhoGDIα negatively regulates actin polymerization and cell migration by inhibiting activity of small RhoGTPases [64], while FBW7 is known to affect degradation of the critical cellular regulators [65, 66]. The co-immunoprecipitation assays between the two proteins in the bladder carcinoma cells showed that FBW7 drives the ubiquitination of and proteasome-dependent degradation of RhoGDIα, resulting in enhanced cell migration. The overexpression of FBW7 reduced RhoGDIα expression and promoted its degradation. Upon the RelA knockdown, however, cells displayed a slower degradation of RhoGDIα, which resulted in its increased protein level, while the opposite effects were seen for FBW7. The FBW7 mRNA level did not change, while the FBW7 degradation rate was markedly increased. These observations suggest that RelA is required for FBW7 stabilization post-transcriptionally, which then promotes RhoGDIα protein degradation and drives migratory activity and invasion. On the other hand, PTEN is inversely coupled with FBW7, where it downregulates the expression of FBW7 at the protein level and inhibits cell migration. Conversely, the loss of PTEN after RelA overexpression stimulates FBW7 mediated- RhoGDIα degradation, attenuates RhoGDIα inhibition on the small GTPases and promotes cell migration [67–69]. Taken together, overexpression of RelA during EMT reduces PTEN expression, which leads to an increased invasive and migratory phenotype of bladder cancer cells through FBW7-mediated degradation of RhoGDIα.

Paxillin is an important adaptor protein that recruits a variety of structural and signaling molecules to focal adhesions, where it coordinates different pathways to elicit changes in cell movement and migration [62]. While a few studies have identified paxillin as a novel interactor of PTEN [70], the mechanisms underlying the upregulation of paxillin and the significance of its coordination with PTEN in cancer progression are just beginning to be revealed. The immunohistochemistry and western blot analysis in colon cancer cells overexpressing PTEN, exhibited decreased paxillin expression at both mRNA and protein levels, while the overexpression of paxillin reduced levels of PTEN and stimulated migration. These data suggested that PTEN and paxillin have antagonistic roles in regulating invasion and migration in colon cancer. Apart from PTEN, it was also found that paxillin contains three NF-κB-binding sites in its promoter region, to which p65 and p50 can bind. This interaction allows NF-κB to regulate the transcription of the paxillin gene, as evident by the inhibition of the paxillin mRNA and protein expression by of PI3K/AKT and NF-κB inhibitors, even in the absence of PTEN [70]. These findings suggest that the expression of paxillin is regulated by a crosstalk between PTEN and the NF-κB pathway. Therefore, PTEN inactivating mutations, which are commonly found in many types of cancer, may lead to overactivation of the PI3K/AKT/NF-κB pathway, which increases paxillin transcription and subsequently results in the invasive properties of cancer cells.

Osteopontin (OPN) acts in coordination with PI3K/AKT and NF-κB pathway to drive the expression of the metastatic-promoting downstream proteins. The overexpression of OPN is found in many cancers and has been implicated in tumor progression and metastasis [71–73]. Particularly, the overexpression of OPN affects cell adhesion, migration, ECM invasion, and proliferation via the interaction with its receptor integrin α_v_β_3_, which induces the production and release of urokinase-type plasminogen activator (uPA) [74]. uPA is a key serine protease that catalyzes extracellular proteolysis and degradation of the extracellular matrix (ECM). The degradation of EMC in turn enhances cancer cell invasion and metastasis, facilitating the metastatic cascade [75–77]. The mechanism by which OPN regulates the expression of uPA involves PI3K/AKT/NF-κB pathway. It was shown that OPN enhances the activities of PI3K, phosphorylation of Akt, as well as the interaction between the phosphorylated Akt and IKKα/β, through α_v_β_3_ integrin [78]. This interaction, in turn, stimulates the downstream IκB/IKK signaling pathway, where IKK phosphorylates and induces degradation of IκBα and results in NF-κB activation. NF-κB binds to a response element in the promoter region of uPA, suggesting that the activated NF-κB has regulatory role in the secretion of uPA [79, 80]. The introduction of either PI3K inhibitor or delta-p85 subunit inhibited α_v_β_3_ integrin-mediated phosphorylation and degradation of IκBα, while silencing of α_v_β_3_ integrin, PI3K, or NF-κB, all resulted in the decreased uPA secretion. Taken together, these results suggest that OPN stimulates NF-κB-mediated uPA secretion through PI3K-dependent Akt phosphorylation, ultimately controlling the motility and invasiveness of breast cancer cells.

In sum, NF-κB serves as a pivotal mediator in the acquisition of an invasive phenotype. It coordinates the activation of multiple downstream effector proteins, such as FBW7/RhoGDIα, paxillin, and uPA, and, among others, is regulated by the PI3K/Akt pathway, which interacts with PTEN and OPN to either suppress or promote cell migration and invasion, respectively. Therefore, therapeutic interventions targeting NF-κB activation may present an attractive solution to reduce cell motility, invasiveness, and the metastatic spread of cancer cells.

#### NF-κB and CXCL12/CXCR4 axis

The increased level of CXCR4 and its canonical ligand CXCL12 (SDF-1α) is frequently found to be elevated in metastatic sites of many cancers, including breast cancer, pancreatic cancer, or cervical/ovarian carcinoma [81]. CXCR4 plays a crucial role in tumor development, proliferation, and metastasis mainly by contributing to the establishment of cancer stem-like cell supporting niches and the chemotactic directing of cancer cells to those microenvironments [82]. NF-κB has been suggested as a transcription factor regulating CXCR4 expression and it was shown that CXCR4 expression is dependent on the activation of the NF-κB pathway in several cancer types [83–85]. For instance, when human breast cancer cells with constitutive NF-κB activity were treated with NF-κB inhibitors, (overexpression of IκBα or parthenolide treatment), the expression of CXCR4 transcripts was notably reduced, followed by a loss of SDF-1α-mediated migration [86]. Conversely, in cells modified to overproduce IL-1, an inducer of NF-κB signaling, enhanced CXCR4 expression and SDF-1α-mediated migration was observed [86]. Electrophoretic mobility shift assays and transient transfection assays revealed presence of NF-κB binding sites on CXCR4 promoter regions, suggesting that NF-κB may directly regulate the transcription of *CXCR4*. Similar results were demonstrated in pancreatic cancer cells, where IL-1 induced NF-κB signaling was coupled with the increased co-expression of another marker of stemness—CD133 on cells that were responsible for the formation of aggressive tumors [87]. Moreover, the gene encoding for IL-1 has been shown to be induced in an NF-κB-dependent manner, establishing a positive feedback mechanism [88]. The overexpression of CD133 was shown to increase the expression of IL-1 receptor and IL-1β, which upregulated NF-κB activity [89] imparting invasive and motile phenotypes. Taken together, these results indicate the role of IL-1-dependent-NF-κB activation in mediating the motility and organ-specific homing of metastatic cancer cells via controlling CXCR4 as well as CD133 expression on the cell surface.

#### NF-κB and matrix metalloproteinases

Metastasis often involves the degradation of the extracellular matrix (ECM) and remodeling of the basement membrane associated with metastasis. Matrix metalloproteinases (MMPs) have been extensively studied in the context of their role in ECM remodeling and degradation [90]. Current evidence suggests a role of NF-κB-signaling in stimulating secretion of cytokines, chemokines or alarmins that directly induce the expression of MMPs, leading to increased cancer invasiveness and metastasis. S100A4 (S100 Calcium Binding Protein A4) is a metastasis-associated protein whose expression was shown to correlate with the metastatic potential and overall clinical prognosis in multiple cancers [91, 92]. Negative correlation between expression of S100A4 and E-cadherin were found with patients with metastatic esophageal cancer. Overexpression of S100A4 led to increased tumor cell invasion, metastasis, and angiogenesis and downregulation of S100A4 reduced VEGF and MMP9 expression. Zhang and colleagues found that expression of S100A4 positively correlated with NF-κB and MMP9, while S100A4 knockdown resulted in significant reduction in the NF-κB activity and MMP9 expression, and decreased invasion [93–95]. These findings suggest that S100A4 controls the motility and invasive potential of cancer cells through the MMP9/NF-κB–based mediation.

#### NF-κB, EMT and cyto- and chemokines

Upregulation of many cytokines and chemokines including CCL27/CCL28-CCR10 [96], CXCL10-CXCR3 [97], IL-8-CXCR2 [98] cytokine/chemokine-receptor pairs has been noted in many cancers. NF-κB signaling is well known to induce expression of pro-inflammatory cytokine/chemokines that contribute to metastatic progression. For example, NF-κB signaling was shown to be linked to IL-5, -15 and -17-induced metastasis. In hepatocellular carcinoma (HCC), elevated levels of IL-17 were associated with worse overall survival and decrease in disease-free survival rates accompanied by the increased incidence of metastasis. It has been reported that NF-κB mediates the downstream signaling of the IL-17A pathway [99], enhancing the activity and expression of MMPs, specifically MMP2 and MMP9 [100–102]. Besides IL-17, IL-5 as well as IL-15 were shown to induce the expression of MMP9 via activating NF-κB and AP-1, causing enhanced cell motility and migration of bladder cancer cells [103, 104]. Taken together, several cytokines including IL-5, -15 or -17 influence the acquisition of an invasive phenotype through NF-κB-stimulated MMP2 and/or MMP9 activities.

NF-κB and JAK/STAT pathways are major inducers/regulators of inflammatory phenotype both at the signaling, cellular and tissue levels. The existing evidence summarized in this review clearly highlights how elevated NF-κB signaling and EMT sustain each other, in an alliance for metastasis and cancer progression. NF-κB induced overabundance of pro-inflammatory cytokines such as TGF*β*, TNF*α*, IL-1, IL-5, IL-6, IL-8, IL-15 or IL-17 activate transcription factors such as Smad, Snail, Twist, and Zeb and induce expression of the mesenchymal markers, such as N-cadherin, fibronectin or vimentin while inhibiting the epithelial cells markers such as E-cadherin, claudin1 or occludin. Pro-inflammatory cytokines/chemokines can also, in a positive feedback loop, reactivate NF-κB and JAK/STAT signaling, closing the cycle of malignant perpetuity. The mechanistic link between NF-κB and STAT signaling and its role in promoting cancer metastasis is out of scope of this review and was summarized elsewhere [1, 105, 106].

### Therapeutic targeting of NF-κB-EMT crosstalk

Our understanding of the signaling pathways driving EMT, together with intricate interactions between cancer cells and cancer immune and non-immune microenvironment is expanding, however we still have not been able to therapeutically meaningfully harness this knowledge. Several strategies have been proposed to target EMT including inhibition of upstream extracellular signals and their respective pathways, inhibiting expression and function of major EMT TFs, targeting mesenchymal cells specifically and inhibiting mesenchymal to epithelial transition (MET) (reviewed in [107, 108]). Much effort has been also directed to explore and develop strategies to effectively target NF-κB pathway. Drugs, that are currently in clinical use to target NF-κB signaling include canonical and non-canonical pathway receptor antagonists (e.g., ibudilast), inhibitors of cascade adapter proteins (e.g., JKB-122, ibubrutinib), inhibitors of IKKα, IKKβ, NEMO or NIK (e.g., IKK module inhibitors CHS-828 or AS-602868) inhibitors of proteasome (e.g., disulfiram, bortezomib). The current therapeutic strategies targeting NF-κB pathway are summarized elsewhere [109]. In this concluding paragraph we aimed to highlight few select compounds that target established links between NF-κB and EMT (Fig. 4). Many signals received from the tumor microenvironment can induce EMT and these include pro-inflammatory cytokines such as TGFß or TNFα. Inhibitors targeting surface receptors or ligand-neutralizing antibodies may represent a useful strategy to inhibit EMT. A specific inhibitor of TGFß receptor kinase -SB-431542 [110], or HIF-1α -IDF-11774 [111] were shown to efficiently suppress tumor-promoting effects in pancreatic cancer cells and colorectal carcinoma, respectively. Inhibition of TNFα-induced IκBα phosphorylation by BAY 11–7082 was reported in glioblastoma cells [36]. PI3K/AKT is one of the pathways involved in regulation of EMT and several drugs targeting PI3K/AKT, such as buparlisib and taselisib, are currently in Phase 3 of clinical trials [112, 113]. Stansel et al. showed that Norcantharidin (NCTD) treatment suppressed nuclear translocation of NF-κB and phosphorylation of IκBα and reduced migration and invasion of hepatocellular carcinoma and colorectal adenocarcinoma cells, via decreasing the expression of MMP9 and uPA [114]. Farnesyl dimethyl chromanol (FDMC) treatment was found to induce decrease in NF-κB signaling and expression of MMP9 resulting in suppression of growth, motility, and invasion in colorectal cancer stem cells [115]. Targeting of the EMT effector transcription factors, including Snail, Slug, Twist, and Zeb1/2, has been recognized as a potential therapeutic strategy in breast cancer cells [116] and non-small cell lung cancer, [117] where targeted inactivation of Snail family suppressed tumor cell proliferation and metastasis. Silencing of Twist also efficiently attenuated metastatic potential in breast tumor cells, reducing the frequency of lung metastases in in vivo mouse study [118]. Metformin, an FDA-approved antidiabetic agent, was found to inhibit the expression of EMT TFs, including Zeb1, and suppressing invasiveness and stemness with improved survival benefits in a mouse model of glioblastoma [119, 120] and is currently under clinical trial Phase 2. It should be however recognized that a critical hurdle of direct targeting of EMT TFs is their complementary and redundant function and existence of efficient feedback mechanisms overcoming their absence. Therefore, targeting multiple EMT TFs should be considered as a potential therapeutic strategy. Targeting the mesenchymal cell markers, such as vimentin or N-cadherin, may be another strategy to eliminate or inhibit existing metastatic growth. Withaferin A (WA), a natural steroidal compound found in the medicinal plant *Withania somnifera*, has been shown to bind to and disrupt vimentin and intermediate filament network, inhibiting EMT in both in vitro and in vivo breast tumors [121]. WA increases apoptosis in a dose-dependent manner, inhibiting cell proliferation by stopping the cell cycle during the G2/M checkpoint. The monoclonal antibody or siRNA targeting of N-cadherin has also been reported to decrease migration, proliferation, and survival of enzalutamide-resistant prostate cancer, therefore representing another potential therapeutic target.

## Conclusions

Tumor promoting inflammation was recognized as a hallmark of cancer in 2011 [122], and the critical involvement of smoldering inflammation in carcinogenesis has been increasingly acknowledged since then Phenotypic plasticity was added to the list of hallmarks in 2022 [123] and involvement of major inflammatory pathway—NF-κB signaling—in regulation of phenotype plasticity has been now widely recognized. EMT is the developmental program that decreases cell–cell adherence allowing cells to acquire migratory properties and features of stem cell-like plasticity both contributing to acquired invasive properties, elevated metastatic and survival potentials. As summarized in this review it seems apparent that NF-κB-induced inflammation is a potent inducer, contributor and regulator of EMT phenotypes. Mechanistic links between NF-κB signaling and steps leading to transition from epithelial to mesenchymal phenotypes exist, but require further detailed mechanistic elucidation to reveal all the involved factors and uncover potential new therapeutic targets. In our search for therapeutics efficiently targeting EMT, it remains imperative to decipher both intracellular mechanisms induced by and driving inflammatory signaling and ensure interpretation of their role acknowledging proper microenvironmental context. Given the available evidence it is reasonable to infer that targeting NF-κB signaling may represent a valuable strategy to target EMT and related mechanisms in cancer.

## Data Availability

Data sharing is not applicable to this article as no datasets were generated or analyzed during the current study.
